# Charge Transport in LDPE Nanocomposites Part I—Experimental Approach

**DOI:** 10.3390/polym8030087

**Published:** 2016-03-16

**Authors:** Anh T. Hoang, Love Pallon, Dongming Liu, Yuriy V. Serdyuk, Stanislaw M. Gubanski, Ulf W. Gedde

**Affiliations:** 1 Division of High Voltage Engineering, Department of Materials and Manufacturing Technology, Chalmers University of Technology, Gothenburg SE-412 96, Sweden; anh.hoang@chalmers.se (A.T.H.); yuriy.serdyuk@chalmers.se (Y.V.S.); 2 Fiber and Polymer Technology, School of Chemical Science and Engineering, KTH Royal Institute of Technology, Stockholm SE-100 44, Sweden; lovep@kth.se (L.P.); donliu@kth.se (D.L.); gedde@kth.se (U.W.G.)

**Keywords:** low-density polyethylene, nanocomposites, dc conductivity, charge carrier mobility, charge transport, trap depth

## Abstract

This work presents results of bulk conductivity and surface potential decay measurements on low-density polyethylene and its nanocomposites filled with uncoated MgO and Al_2_O_3_, with the aim to highlight the effect of the nanofillers on charge transport processes. Material samples at various filler contents, up to 9 wt %, were prepared in the form of thin films. The performed measurements show a significant impact of the nanofillers on reduction of material’s direct current (dc) conductivity. The investigations thus focused on the nanocomposites having the lowest dc conductivity. Various mechanisms of charge generation and transport in solids, including space charge limited current, Poole-Frenkel effect and Schottky injection, were utilized for examining the experimental results. The mobilities of charge carriers were deduced from the measured surface potential decay characteristics and were found to be at least two times lower for the nanocomposites. The temperature dependencies of the mobilities were compared for different materials.

## 1. Introduction

Polyethylene (PE) has been widely used as cable insulation material thanks to its low electrical conductivity. Despite the successful application of this material for high voltage alternating current (HVAC) cables, a number of challenges has been encountered in its use in high voltage direct current (HVDC) counterparts [1]. Unlike the case of ac stress, the electric field distribution under dc stress is governed by material’s dc conductivity. This parameter is dependent on both electric field and temperature. As cable insulation usually operates at a temperature gradient, electric field distribution inside the insulation bulk is a complex function of material properties and radial position [2]. Space charge accumulation and, hence, local field enhancements are usually observed inside HVDC insulation, which may stimulate accelerated ageing process [3].

The forecasted growth in worldwide demand for electrical power energy and the requirement of longer transmission distances are the incentives for designing extruded HVDC cables that should reliably work at high rated voltage, e.g., up to 1 megavolt (MV), and have high power transmission capability, up to several gigawatts (GW). For such HVDC cables, the problem of field enhancement and space charge accumulation must be effectively solved and the most important requirement should be an extremely low dc conductivity of its insulation. A promising approach for dealing with this task is the application of nanotechnology, which allows for creating new materials with superior properties by adding a small amount of nanoparticles [4]. In case of insulation for HVDC cables, even though the semi-crystallized PE can be considered itself as a natural nanometric dielectric [5], the introduction of nanoparticles brings about a variety of advantages. In particular, significant reduction in dc conductivity, negligible space charge accumulation in the bulk as well as an increased dielectric strength have been observed in PE nanocomposites in comparison to unfilled counterpart [6,7,8,9,10]. The improvements in properties of nanomaterials have been attributed to the formation of interfacial regions between nanofillers and base polymer which are characterized by enormously high ratio of surface area to volume [6,11].

Although many investigations on PE nanocomposites have been reported recently, the transport of charge carriers contributing to their dc conductivity is not fully understood yet. In this context, a model describing transport of charge carriers in PE with and without nanofillers is highly desirable for analyzing the role of nanofillers in conduction processes. To formulate such a model, consistent input parameters need to be provided, in particular, mobilities of charge carriers in the materials. The latter have been studied extensively for pure PE for which it has been found to be dependent on both electric field and temperature [12,13,14,15], while information is very limited in case of PE-based nanocomposites.

In the present investigation, we attempt to address several aspects in the above defined gap of knowledge. Both experimental and simulation techniques are utilized and the obtained results are reported in two articles. In the first one here, we present measured charging currents and surface potential decay (SPD) characteristics obtained at various temperatures on two types of low-density polyethylene (LDPE) nanocomposites as well as on unfilled material. The most important parameters governing the conduction processes in the materials are deduced, namely the mobilities of charge carriers and energy distributions of traps. In the second paper, a model of charge transport is developed for LDPE with and without nanofillers. Materials’ parameters attained from the measured data are used as input for the model whereas the measured current characteristics are utilized for validation of the simulated results.

## 2. Materials and Methods

### 2.1. Samples

Nanocomposites were manufactured using two types of uncoated metal-oxide nanofillers, namely alumina (Al_2_O_3_) and magnesia (MgO). The Al_2_O_3_ particles had spherical shape with an average diameter of 40 nm, whereas the MgO nanoparticles were in rounded hexagonal shape with an average size of 66 nm and a thickness of 10–20 nm. The nanoparticles used are characterized by narrow size distributions and high purity [16,17]. For preparing nanocomposites, a certain amount of nanoparticles and Irganox 1076 (used as antioxidant) were dispersed in heptane solvent and the suspension was added into LDPE powder. The obtained mixture was then shaken for 1 h and dried in an oven at 80 °C to evaporate all the solvent. Finally, the dry mixture powder was compounded by thermal extrusion at 150 °C in 6 min. The obtained materials were later on pressed to form 80 µm thick films that have square shape with a side of 65 mm. The prepared samples were then kept in a desiccator for preventing the intake of moisture from laboratory air.

To study the influence of filler content on material properties, two nanocomposites filled with Al_2_O_3_ at 1 and 3 wt % as well as five types of MgO-filled materials with filler content of 0.1, 1, 3, 6, and 9 wt % were prepared. All the materials contained the antioxidant at 0.02 wt % for avoiding degradation by oxidation. Both nanofillers are evenly distributed in LDPE matrix, as presented in [16] for LDPE/Al_2_O_3_. In case of LDPE/MgO at high filler contents (6 and 9 wt %), clustered and agglomerated particles were observed. While the clusters only consist of a couple of nanoparticles and are less than a micrometer in cross-section, the agglomerates can be several micrometers large and built up of thousands of nanoparticles. Detailed information on the particle distance and the degree and size of agglomerates is presented in a separate publication [17].

### 2.2. Conductivity Measurements

**Figure 1:**
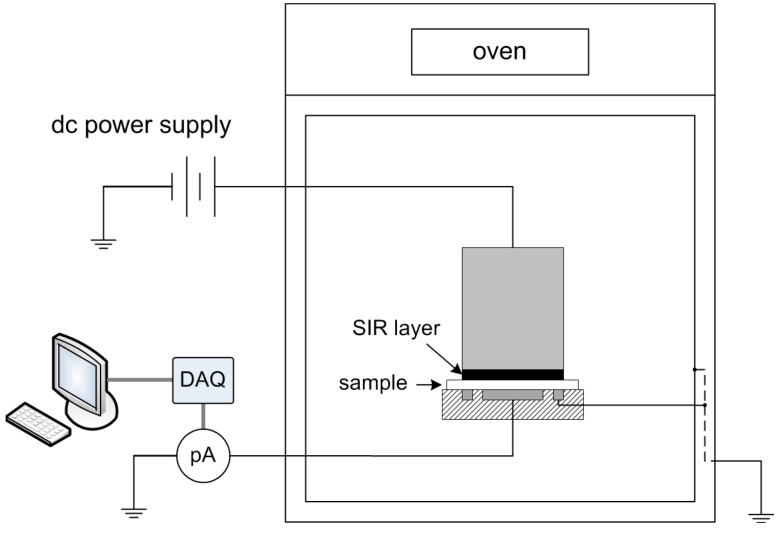
Schematic illustration of the test setup for conductivity measurements. DAQ, denotes data acquisition card and pA, picoammeter.

Measurements of dc conductivity were carried out at applied electrical field of ~30 kV/mm. The test setup is shown in Figure 1. The dc test voltage was generated by a Glassman power supply (model FJ60R2) and the current was measured using an electrometer Keithley 6517A (Tektronix Inc., Beaverton, OR, USA). The experiments followed a standard procedure [18] by using a three-electrode system, of which the high voltage electrode was a stainless steel cylinder with a diameter of 45 mm, the current measuring electrode was 30 mm in diameter, whereas the guard ring allowed for eliminating surface currents. A good contact of the high voltage electrode to the sample was provided by placing between them a layer of conducting silicon rubber (SIR) (Elastosil 570/70 from Wacker Chemie AG, Munich, Germany; dc conductivity of 28 S/m). The use of the SIR electrode in the measurements resembles the operating conditions of cable insulation that is always in contact with a semiconducting layer.

The measurements were conducted at isothermal conditions (room temperature ~20–22 °C, 40 °C, and 60 °C). The latter two temperature levels were reached by placing the electrode system with inserted sample inside an oven. In this case, the metallic walls of the oven were grounded constituting a shielding box for avoiding electromagnetic disturbances. Thermal equilibrium at a predefined elevated temperature was attained by keeping the setup inside the oven for ~2 h prior to each test. Thereafter, a dc voltage of 2.6 kV was applied to the high voltage electrode for 4 × 10^4^ s (*i.e.*, ~11 h) and the current was recorded. Each test was repeated 2–3 times for checking the reproducibility of the results. The measured data were collected and stored in a personal computer via a data acquisition card (DAQ).

### 2.3. SPD Measurements

**Figure 2:**
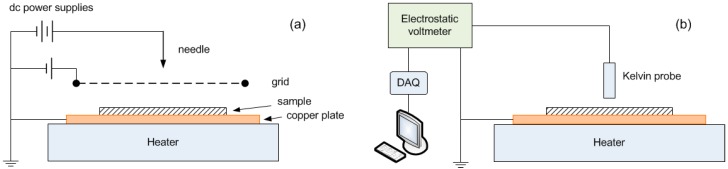
Schematic illustration of the setup for corona charging (**a**); and surface potential decay measurements (**b**).

The experimental setup for SPD measurements is schematically illustrated in Figure 2. During the experiment one side of the film samples remains in contact with a grounded copper plate, while the other side is initially exposed to corona charging in air for 10 s. The corona is generated in a triode electrode system [19], which consists of a needle and a grid electrodes connected to dc voltage sources. The use of the grid electrode allows for improving uniformity of the deposited charges as well as for controlling the level of surface potential on the charged surface. The magnitude of the voltage applied to the grid was selected so that the initial electric field induced in the samples by the deposited charges was close to the electric field applied during the conductivity measurements. The potential induced by deposited surface charges was measured by means of a non-contact technique [20] using a Kelvin probe placed above the sample surface. The probe was connected to an electrostatic voltmeter (Trek model 347B). The positions of the corona triode and the probe were controlled by a positioning system. Surface potential was continuously monitored at the center of the sample and potential distribution was regularly checked by scanning the surface through the center position. The data were stored for further analyses by using LabVIEW software (National Instruments, Austin, TX, USA).

The SPD measurements were conducted for two nanocomposites filled with 3 wt % of nanoparticles as well as on the reference material at three temperatures, as for the conductivity measurements. The sample heating was realized by means of a hot plate on which the grounded copper plate rested. Prior to each test at elevated temperatures, the sample was preconditioned at a targeted temperature for ~4 h, thus assuring that homogeneous temperature distribution is achieved in the tested thin film.

**Figure 3:**
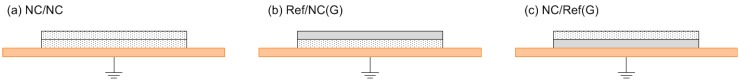
Multilayered sample structures used in SPD measurements: (**a**) NC/NC; (**b**) Ref/NC(G); and (**c**) NC/Ref(G). Ref and NC denote respectively the reference LDPE and LDPE/Al_2_O_3_ 3 wt % nanocomposite whereas index (G) indicates the layer in contact with the grounded copper plate during the test.

The SPD measurements were also conducted on multilayered sample structures. For this, three specimen configurations were used (NC/NC, Ref/NC(G), NC/Ref(G)), as illustrated in Figure 3. The initial electric field induced inside the insulation was kept at the same level as for the measurements on single-layered samples by increasing the voltage applied to the grid electrode. Since surface potential exceeding 3 kV should be detected, a Trek electrostatic voltmeter model 341B was utilized which allowed for measurements up to 20 kV. The tests were conducted at room temperature only by following the same experimental procedure as described earlier. Each SPD measurement was performed 2–3 times for checking the repeatability of the results.

## 3. Results and Discussion

### 3.1. Material DC Conductivity

Preliminary measurements showed that the addition of a small amount (0.02 wt %) of antioxidant into LDPE did not cause noticeable variation in material dc conductivity. LDPE doped with antioxidant is therefore utilized as a reference material throughout this study.

**Figure 4:**
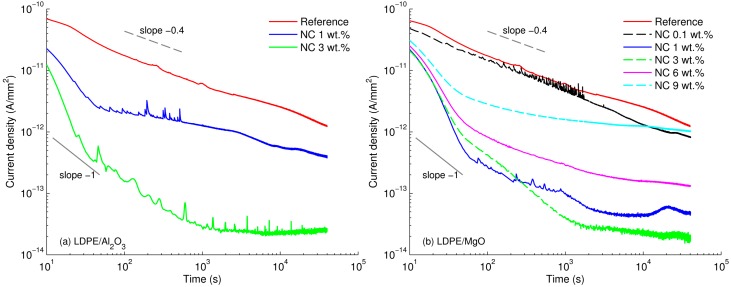
Densities of charging currents as functions of time measured at 60 °C for reference LDPE and both nanocomposites (Al_2_O_3_ (**a**) and MgO (**b**)).

Figure 4 illustrates time variations of the density of the measured currents at 60 °C, which can be represented by power functions with various values of factor *n*

(1) $$j\left(t\right)\propto t^{-n}$$

As seen, the currents through the reference LDPE and LDPE/MgO 0.1 wt % materials decreased gradually and their time dependences exhibit a straight line (in the log-log scale) with a single slope *n* ≈ 0.4. It is notable that these currents do not reach a steady state during the measuring time (~11 h) used in the present study. Adamec and Calderwood [21] suggested that such slowly decaying currents can be attributed to the effect of space charge build-up in the bulk rather than to slow dipole orientation. Their hypothesis has been supported by the fact that the discharging current was remarkably lower than the charging counterpart, which indicated insignificant dipole depolarization [21]. Note that even though PE is well-known as a non-polar polymer, dipolar moieties such as impurities or by-products of oxidation may still exist in the material, resulting in the apparent polarization.

The shape of the recorded current traces changes significantly in cases of nanocomposites with filler content of 1 wt % or higher. A knee point at ~50–70 s after the voltage application can be seen in the characteristics of these materials. Within the initial 50–70 s, the current decayed rapidly with the slope *n* exceeding 1 that is more likely due to the slow polarization as suggested in [21]. Thereafter, as the polarization process ceases, the conduction current becomes prevailing and the power factor *n* in Equation (1) is getting closer to zero that corresponds to a steady state, *i.e.*, dc conduction mode. In the following, the quasi-steady state conduction current observed at ~4 × 10^4^ s is used for comparisons. Overall, the measured currents are commonly lower for the nanocomposites as compared to the reference material, indicating a weakening of the charge transport. Thus, for LDPE/Al_2_O_3_ nanocomposite at the nanofiller content of 3 wt % the current is reduced by almost two orders of magnitude. A less pronounced reduction is found in case of 1 wt % of nanofiller load. For LDPE/MgO nanocomposite, a significant drop is also exhibited at filler loading of 3 wt %, whereas lower (0.1 wt %) or higher (9 wt %) amounts of this nanofiller do not result in a noticeable change of the property.

**Figure 5:**
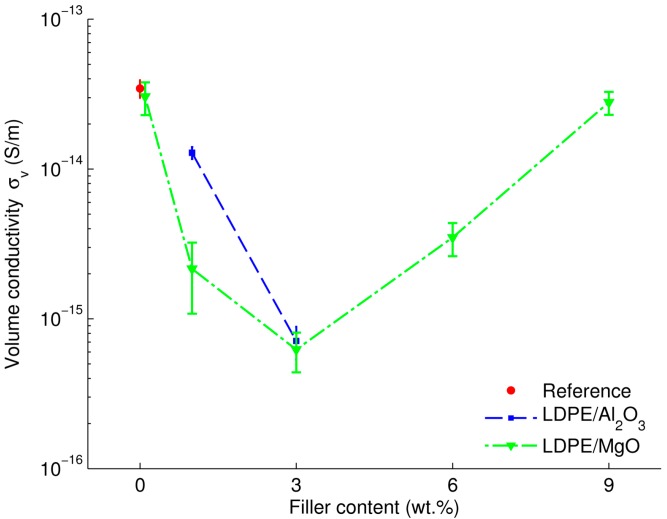
Dependence of dc conductivity (at 60 °C) of the studied nanocomposites on filler content.

The materials’ dc conductivities calculated by utilizing the charging currents at 4 × 10^4^ s are shown in Figure 5. For LDPE, the outcome is in good agreement with data reported in literature, e.g., [8]. For the LDPE/Al_2_O_3_ nanocomposite, the reduction in dc conductivity seems to be proportional to the filler content up to 3 wt %. For the LDPE/MgO nanocomposite, a threshold-like behavior can be noticed at ~3 wt %. As seen from the plot, after reaching this point, further addition of nanoparticles causes a negative effect, *i.e.*, the dc conductivity increases remarkably with higher filler loading that can be explained by a formation of agglomerations of nanoparticles in the base material [10]. The obtained results for the LDPE/MgO nanocomposite are in line with earlier reported investigations [9,10] where a decrease in electrical conductivity in more than one order of magnitude and a threshold of filler loading at ~2 wt % were observed.

**Figure 6:**
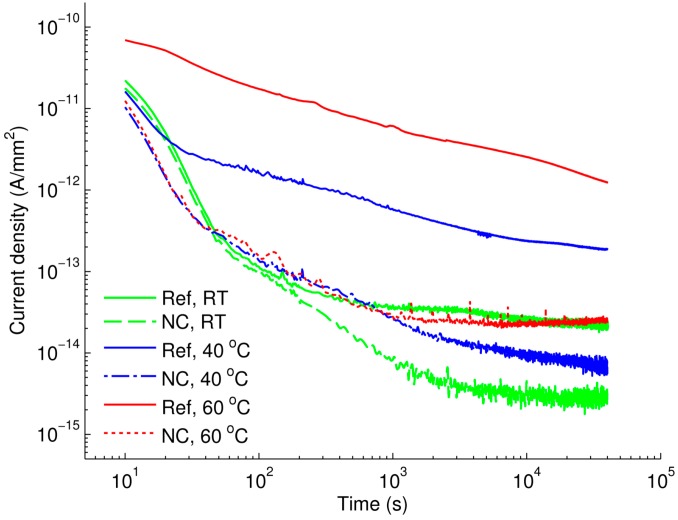
Densities of charging currents as functions of time measured at room temperature (RT) ~20–22 °C, 40 °C, and 60 °C for the reference LDPE (Ref) and 3 wt % LDPE/Al_2_O_3_ nanocomposite (NC).

Further investigations focused on analyzing the temperature dependence of dc conductivity. The study was carried out on the nanocomposites showing the greatest reduction in dc conductivity, *i.e.*, the materials with filler loading of 3 wt %. Since the results obtained for the two nanocomposites were quite similar, only the current densities measured for LDPE/Al_2_O_3_ are presented in Figure 6 and are compared with those for unfilled LDPE. Results for LDPE/MgO nanocomposite can be found in Figure S1 of the supplementary materials. It is noteworthy to mention that the time dependence of the current density measured at room temperature on the reference LDPE was in good agreement with the corresponding result reported in [22]. As it is seen in Figure 6, the reduction in the current density associated with the introduction of nanofillers is the most remarkable at 60 °C, whereas it is lower at room temperature.

**Figure 7:**
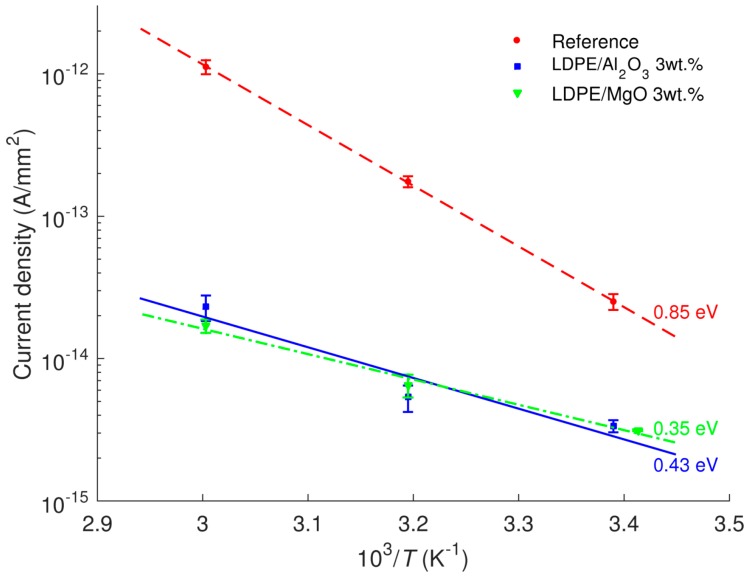
Temperature dependences of current densities at 4 × 10^4^ s of LDPE and its nanocomposites. The calculated activation energies are indicated.

The current densities obtained at 4 × 10^4^ s as functions of the reciprocal of the absolute temperature are shown in Figure 7. The activation energies *W*_a_ for the studied materials can be derived by assuming Arrhenius type of temperature dependence

(2) $$J_{C}\left(T\right)=J_{0}\exp\left(-\frac{W_{a}}{kT}\right)$$

where *J*_C_(*T*) are measured current densities at various temperatures, *J*_0_ is a constant value, *k* is the Boltzmann constant, and *T* is absolute temperature. The calculated values are indicated in the figure. The activation energy is higher for the reference LDPE as compared to both the nanocomposites. The outcome therefore suggests that at temperatures higher than 60 °C, the reduction in dc conductivity due to the introduced nanoparticles would be even more pronounced and the associated charge transport is much more suppressed.

### 3.2. SPD on Single-layered Insulation

**Figure 8:**
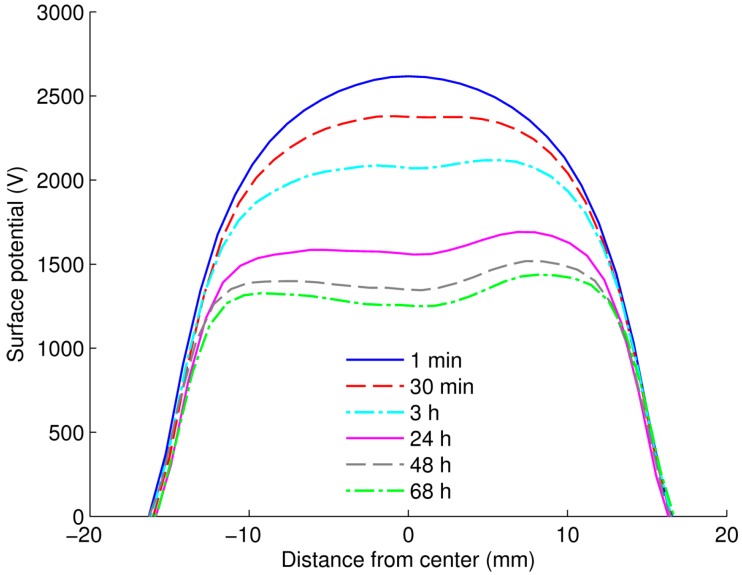
Distribution of surface potential during potential decay measurement on LDPE/Al_2_O_3_ 3 wt % nanocomposite at 60 °C.

A distribution of surface potential measured on LDPE/Al_2_O_3_ nanocomposite is illustrated in Figure 8, which is typical results obtained at all considered temperatures on both materials. The initial surface potential distribution is relatively homogeneous in the center of the sample and the profile remains generally preserved during the measurements. A lateral spreading of the surface potential/charge is not noticed, indicating a negligible contribution of surface conduction to the decay process. As the Kelvin probe was always kept above the center of sample surface, zero electric field was maintained in the air gap between the surface center and the probe. Thus, neutralization by ionic species from air was to great extend prevented [23]. As a consequence, the decay is believed to be mainly caused by processes in the insulation bulk.

**Figure 9:**
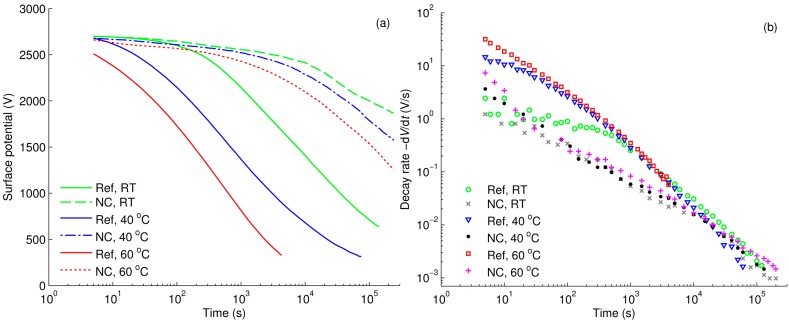
Measured surface potentials (**a**); and calculated decay rates (**b**) for reference LDPE (Ref) and LDPE/Al_2_O_3_ 3 wt % nanocomposite (NC) at different temperatures.

The decays of surface potentials on Al_2_O_3_-nanofilled and reference LDPE are compared in Figure 9a, where zero time corresponds to the end of corona charging. The initial potentials were recorded at ~4–5 s afterwards and they are close to the value of grid potential, except for reference LDPE at 60 °C. The decay appears to be considerably slower for the nanofilled LDPE as compared to the reference LDPE, especially at higher temperatures. Since SPD is attributed to the conduction through the bulk, *i.e.*, the transport of charge carriers within the material driven by the field of deposited surface charges, the experimental data imply significant limitation of charge transport due to the introduced nanoparticles.

The decay rates of the surface potentials (Figure 9b) can be represented as power-law functions of time. As temperature increases, a remarkable distinction in decay rates is observed at the initial stage, for the reference LDPE within the first 100 s of the decay. Note the initial drop of the potential was so high at 60 °C that the first measured potential point was ~200 V lower than the grid potential. However, after 10^3^ s, the decay rates became similar for the samples exposed to different temperatures. This observation should not be misinterpreted as indicating a similarity in charge transport process. It is due to the difference in the magnitude of electric field induced in the material at certain time, in particular, the highest field strength presented in the sample subjected to the lowest temperature, so that the apparent decay rates are comparable. As the main features in the SPD characteristics are similar for both nanofilled LDPE, measured results on LDPE/MgO nanocomposite are presented in Figures S2,S3 in the supplementary materials.

### 3.3. SPD on Multilayered Samples

Measurements of surface potential decay on multilayered samples were conducted with the aim of revealing contributions of different processes to the decay in the considered conditions. Before presenting and discussing experimental results, we would like to provide a brief summary of physical processes that may take place during SPD measurements in the bulk and at interfaces of the flat samples depicted in Figure 2 and Figure 3. First of all, high electric field induced by ionic charges created by corona and deposited on sample surface may stimulate charge generation processes in insulation bulk according to, for example, Poole-Frenkel mechanism. Secondly, electronic charges can be injected into the bulk from the metal-insulation interface [24]. Furthermore, other processes may occur at the air-insulation interface. A commonly used assumption is that deposited charges are trapped in deep surface traps and their release yields the decay of measured surface potential [25]. This surface controlled potential decay process is referred to as surface de-trapping mechanism. On the other hand, Baum *et al.* [26] suggested an electron transfer process between the deposited ionic charges and the surface states that results in the appearance of either holes or electrons in the latter, depending on the polarity of corona source. In other words, charges are apparently injected into insulation at the air-insulation interface. These injected charge carriers participate in the transport driven by the induced electric field that is reflected in the decaying surface potential. This hypothesis is commonly referred as the charge injection and transport model and has been used to explain results of SPD measurements in a variety of works, e.g. [13,14,27]. Even though an electric field exceeding 10^7^ V/m may be considered as sufficient enough for charge injection, a threshold value corresponding to its onset is not clearly indicated in literature. It is noteworthy to mention that in general both the surface de-trapping and charge injection mechanisms may take place during SPD. The former seems to be dominating on thin dielectric layers of a few µm [25,28] in which extremely deep surface traps exist, whereas the latter is considered to be prevailing on relatively thicker samples, usually of a few tens of µm [13,14,26], provided that the induced field is strong enough.

**Figure 10:**
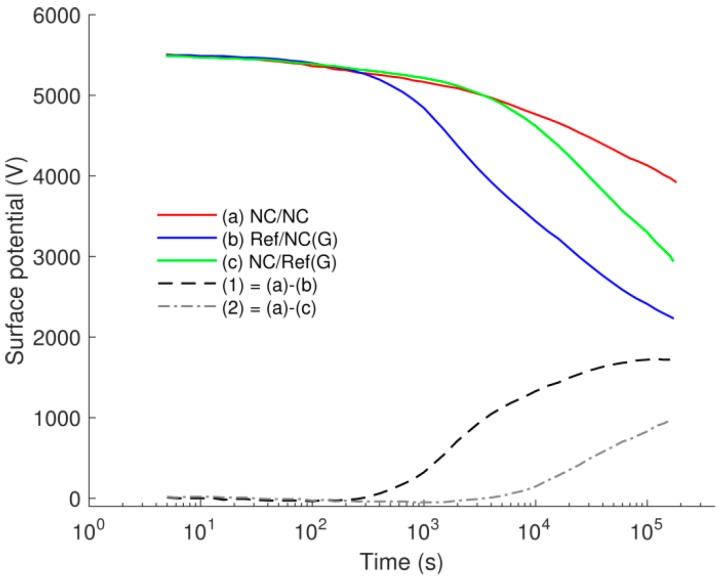
Surface potential decay on multilayered samples. Decay curves (a)–(c) are respectively obtained on samples (a)–(c) illustrated in Figure 3. Curve (1) is a difference in surface potential measured on samples (a) and (b), whereas curve (2)—is the difference for samples (a) and (c).

Results of SPD measurements on multilayered samples are presented in Figure 10. They exhibit a resemblance in potentials measured within the first 200 s. Thereafter, the fastest decay can be observed on Ref/NC(G) sample, whereas the slowest one—on NC/NC sample. If charge generation in the bulk, e.g., by Poole-Frenkel mechanism, is assumed to be the sole contributor to the decay, the same amount of electrons and/or holes would arise in conduction and/or valence bands due to excitation from donors and acceptors in samples Ref/NC(G) and NC/Ref(G). This eventually leads to similar potential decay on these specimens. As the latter is contradictory to the experimental results, this assumption can be ruled out. Combination of charge generation in the bulk and charge injection at the metal-insulation interface is also unlikely the dominating processes as this would lead to a faster decay on NC/Ref(G) sample than on Ref/NC(G).

By comparing decay curves (a) and (c), one can observe that the reference LDPE as the bottom layer in NC/Ref(G) sample slightly alleviates the decay as compared to the NC bottom counterpart in NC/NC sample. The difference in the decay is thus most likely due to an enhanced charge injection from the grounded copper plate into the LDPE layer. This can be related to the difference in dc conductivity measured on these materials. In contrast, significant difference in potential decays was obtained on samples NC/NC and Ref/NC(G) (the decay curves (a) and (b) in Figure 10). Since charges injected from the grounded copper plate were strongly prevented in both structures by the highly resistive NC bottom layer, the observed difference should mainly be attributed to the intensity of charge (hole) injection into the top layers of either NC or LDPE.

**Figure 11:**
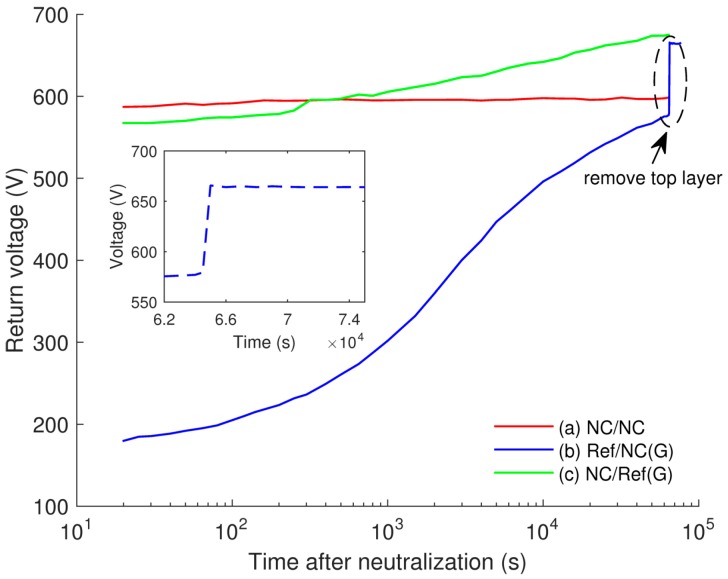
Return voltages measured after short-circuiting multilayered samples for 10 s at the end of SPD measurement. The inset shows the measured potential before and after removal of the top layer of sample Ref/NC(G).

**Figure 12:**
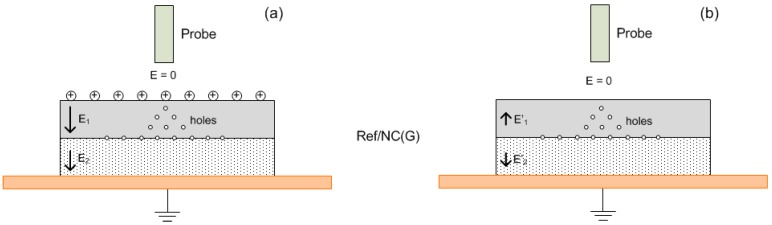
Schematic illustration of charge distribution and electric field (**a**) prior to; and (**b**) immediately after short-circuiting the Ref/NC(G) sample.

The possibility of injection at the air-insulation interface can be supported by appearance of return voltage [29] obtained in our experiments after short-circuiting the multilayered samples at the end of the SPD tests. The short-circuiting was done by placing a metallic electrode that was connected to ground on the sample surface for 10 s. The return voltage is understood here as a potential build-up after temporarily short-circuiting the previously charged object. In the measurements, the return voltages were built up on all the three considered samples (Figure 11), but it was most considerable on Ref/NC(G) sample. According to a simplified model proposed in [30], the appearance of return voltage can be explained by movement of charge carriers back to the surface. A schematic distribution of charges on the surface and in the bulk of Ref/NC(G) sample is proposed in Figure 12. Holes that are initially injected into the top layer and transported in the bulk accumulate at the interface between reference LDPE and nanocomposite as well as in the insulation bulk (Figure 12a). The proposed charge distribution resembles the results of space charge measurements reported in [31]. It is thus postulated that the injection of electrons into the bottom layer is strongly impeded due to its low dc conductivity and presence of these electrons is not shown in the figure. After neutralization, ionic charges on insulation surface cease and the electric field within the top layer is mainly created by the hole space charges. This corresponding induced field should be strongly reduced as compared to that before neutralization and its direction is altered (Figure 12b). The charge transport driven by this weak field requires more time to reach equilibrium distribution inside the considered sample. As an illustration, the measured return voltage did not reach a steady state level even after 18 h. Removal of the top LDPE layer led thereafter to an abrupt increase of the measured return voltage from ~570 V to ~660 V, as shown in the inset in Figure 11. This implies that the measured return voltage would increase further if the top layer was not removed. In contrast, the return voltage build-up for NC/NC sample was very small (~10 V), which can be explained by the reduced charge transport in the nanocomposite. For NC/Ref(G), the return voltage was ~100 V, which is most likely associated with the transport of negative space charges in the bottom layer of LDPE.

By comparing the results of SPD measurements on samples Ref/NC(G) and NC/Ref(G), see decay curves (b) and (c) in Figure 10, one can claim that the contribution of injected positive charges to the decay outweighed by far that of the injected negative charges. The experimental results are also consistent with the hypothesis that positive holes dominate the charge transport in LDPE [32]. In complementary, it can be suggested that this feature is preserved in LDPE nanocomposites, even though the introduction of nanofillers strongly weakens the transport of both the injected holes and electrons.

**Figure 13:**
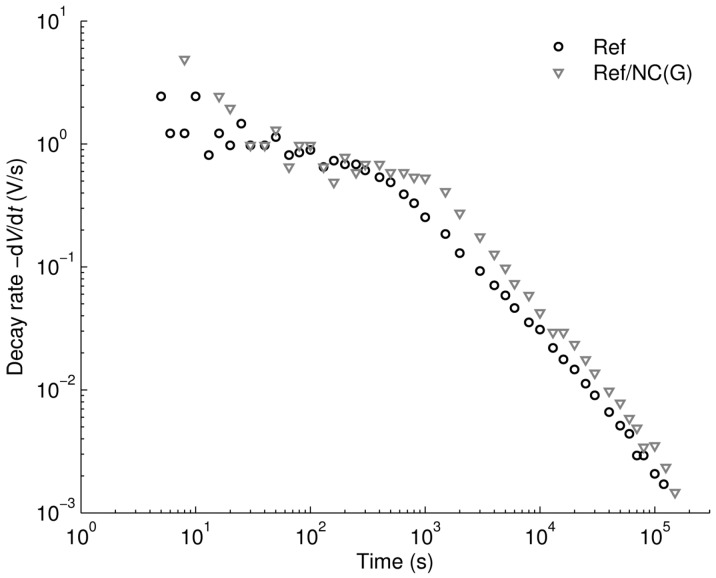
Decay rate of surface potential on reference LDPE and Ref/NC(G).

An additional interesting outcome from the study is presented in Figure 13, where the decay rates of surface potential measured on single-layered reference LDPE sample and on multilayered Ref/NC(G) are compared. As can be seen, the results for both cases are very similar and show a knee point at ~10^3^ s, where the slope of the decay rate characteristic changes. The knee point can be attributed to the arrival of the charge carriers injected at the air-insulation interface [29] to the counter electrode or to the materials’ interface. Consequently, the corresponding time ~10^3^ s may be treated as a transit time of injected holes in the single-layered LDPE sample or that in the top layer of the Ref/NC(G) sample.

In summary, the results of SPD measurements on multilayered samples provide convincing evidence that bipolar charge injection takes place under the experimental conditions of this study, though the positive charge carriers (holes) dominate the transport in reference LDPE. This conclusion can also be extended to the case of single-layered sample, as a similar magnitude of the initial field strength is induced inside the material bulk.

### 3.4. Mobility of Charge Carriers Deduced from SPD Measurements

Dated back to the 60 and 70 s of the last century, the main interest of SPD measurements was related to explanation of the crossover phenomenon that was first reported by Ieda *et al.* [33]. The crossover phenomenon is referred to a faster decay process recorded on dielectric materials being charged to a higher surface potential, so that decay curves cross over each other if their initial surface potentials are different. The crossover phenomenon can be attributed to the non-linear behavior of the dielectric exposed to high electrical field. One of the models that provides a reasonable explanation for this was developed by Sonnonstine and Perlman in 1975 [27]. It accounts for injection of charge carriers from the air-insulation interface and their transport in the bulk of dielectric. By using the model, the authors derived effective mobility of charge carriers [34] which is proportional to the initial decay rate and inversely proportional to the square of the initial field:

(3) $$-{\left(\frac{dV}{dt}\right)}_{t=0}=\frac{\mu }{2}{{\left(\frac{V}{L}\right)}^{2}}_{t=0}$$

Thus, this method can be applied to attain mobility of holes in reference LDPE where they are injected from the air-insulation interface and dominate in charge transport as discussed in Section 3.3 (the same seems to be also valid for the studied LDPE nanocomposites). An alternative way relies on the observation of the knee point in the decay rate characteristics, being attributed to the transit time of charges through the bulk [29]. However, since a knee point is only discernible for reference LDPE at room temperature (Figure 9b), this method is solely applicable in this particular case.

Values of the effective mobility of holes in LDPE at room temperature calculated by the two described methods are respectively 4.2 × 10^−15^ and 2.4 × 10^−15^ m^2^/(Vs), which can already be considered as fairly agreeing with each other. The hole mobility in PE within a range (1−5) × 10^−15^ m^2^/(Vs) at electrical field strength of (2−4) × 10^7^ V/m was obtained in numerous investigations of surface charge decay [13,14,26,35], space charge measurements [32] as well as measurements of transient current [36]. Either slightly lower [15] or marginally higher [34] values of mobility can also be found in literature. As for electron mobility, it has been reported to be few times up to one order of magnitude higher than for the holes [13,32].

**Figure 14:**
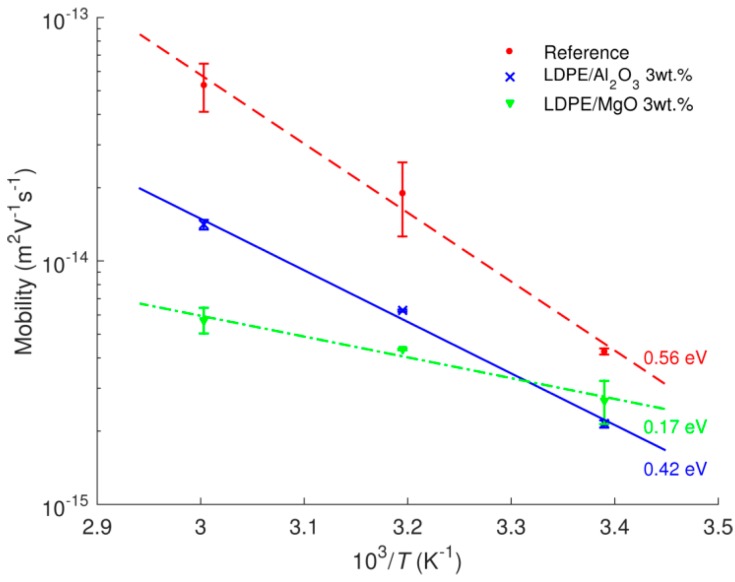
Temperature dependences of charge carrier (hole) mobility derived based on Sonnonstine and Perlman model.

The results obtained by the procedure proposed by Sonnonstine and Perlman are illustrated in Figure 14. As seen, the effective mobility of holes is lower for the nanocomposites, and this difference exaggerates at higher temperatures. The reduction in charge mobility in nanofilled materials has also been reported in [7,10]. Lewis [37] has recently explained the reduced mobility of charge carriers in nanocomposites by modifications introduced by nanoparticles to the energy structure of the amorphous phase in semi-crystalline PE. The author attributes the hole transitions in unfilled PE to tunneling between donor and acceptor sites in the interfacial regions of the amorphous phase [38]. Thereby, the presence of nanoparticles modifies the height of the energy barrier for tunneling as well as the tunneling distance. As a consequence, the time for hole transitions is lengthened and charge carrier mobility decreases [37].

The activation energy for carrier mobility *W_a_*_µ_ can be obtained by using Arrhenius dependence similar to Equation (2):

(4) $$\mu (T)=\mu_{0}\exp\left(-\frac{W_{a\mu }}{kT}\right)$$

where µ(*T*) represents charge mobility at temperature *T* and µ_0_ is a constant. The respective calculated activation energies are indicated in Figure 14 and provided Table 1. The latter also provides activation energies derived earlier from the dc conductivity measurements. It is noteworthy to observe that the values of activation energies for LDPE/Al_2_O_3_ nanocomposite obtained by both methods are close to each other. However, this is not the case for reference LDPE and LDPE/MgO nanocomposite. By recalling the expression for the current density *j*

(5) $$j=qE\sum_{i}n_{i}\mu_{i}$$

where *q* is elementary charge (*q* = 1.6 × 10^−19^ C), it is suggested that for the latter two materials, the density *n_i_* of mobile charge carriers may also increase with temperature.

Conduction in PE has been discussed in a variety of works. A short summary provided in [39] shows that different conduction mechanisms may dominate in the material, depending on experimental conditions. Although the presented values of activation energy vary broadly, a range of 0.84–1.2 eV appears commonly and the activation energy gained in this study is close to the lower limit of the indicated interval. On the other hand, not much information can be found on the activation energy level for conduction in PE nanocomposites. The lower values of activation energy for the nanofilled LDPE presented here indicate that the conduction processes are less temperature-dependent, which would lead to less pronounced field enhancement and space charge accumulation in HVDC cable insulation, which is a positive aspect brought about by the nanofillers.

### 3.5. Plot of −tdV/dt vs. log(t)

The plot of −*t*d*V*/d*t* *vs.* log(*t*) has been widely employed for representing data of SPD measurements as it may reveal information about charge trapping and transport in disordered solids. As pointed out in [28], for the case of exponential potential decay $V=V_{0}\exp(-t/\tau )$, the peak in this plot corresponds to the characteristic time *τ*. The exponential potential decay is however rarely observed in reality. The peak of the curve –*t*d*V*/d*t* *vs*. log(*t*) for a general decay shape can be related either to an average transit time of charge carriers, provided that charge injection takes place, or to an average residence time of charges in trapping sites in the case the surface de-trapping dominates [28]. The later hypothesis has been linked to the demarcation energy model [25], according to which the release of charges from traps at particular time *t* yields potential decay d*V*/d*t*, and hence, the plot of −*t*d*V*/d*t* *vs.* log(*t*) shows a dynamic border between the filled (deeper) and the emptied (shallower) localized states. Thus, the energy depth of traps *E*_t_ is determined by time *t* that charges spend in them:

(6) $$E_{t}=kT\ln\left(\nu_{0}t\right)$$

where *ν*_0_ is the attempt-to-escape frequency. Since −*t*d*V*/d*t* is proportional to the trap density and time *t* is related to the trap depth, the characteristic −*t*d*V*/d*t* *vs.* log(*t*) provides the image of trap energy distribution in considered materials.

**Figure 15:**
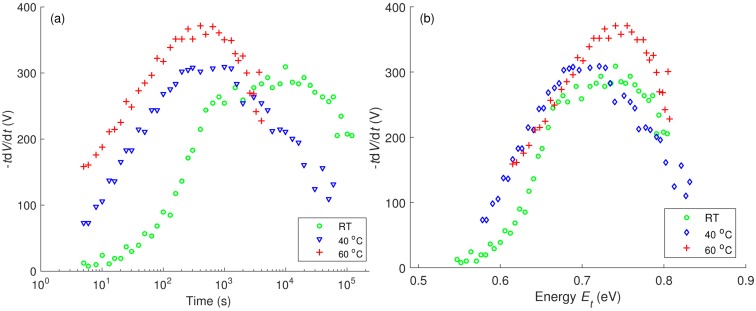
Plots −*t*d*V*/d*t* *vs*. log(*t*) (**a**); and −*t*d*V*/d*t* *vs.* *E_t_* (**b**) obtained at different temperatures for reference LDPE.

The plots of −*t*d*V*/d*t* *vs.* log(*t*) for reference LDPE obtained at different temperatures are presented in Figure 15a. At room temperature, the characteristic shows a broad peak with a shoulder. The time corresponding to the shoulder (~10^3^ s) is close to the transit time of charge carriers, whereas the peak time (~10^4^ s) is longer and appears to be the average dwelling time of charges in deep traps. As temperature rises, the peaks become narrower and the shoulder less pronounced. One can derive the value of the attempt-to-escape frequency *ν*_0_ by using the characteristics of –*t*d*V*/d*t* *vs.* log(*t*) obtained at different temperatures with an assumption of temperature-invariant distribution of trap energy [25]. The calculation provides a value *ν*_0_ ≈ 4 × 10^8^ s^−1^ and the trap depth at maximum density is *E*_t_ ≈ 0.72 eV (Figure 15b). Both the derived values appear to be much lower than the commonly accepted parameters (ν_0_ in order of 10^12^ s^−1^ and *E_t_* ≈ 1.0 eV). This discrepancy might be attributed to the fact that the decay is controlled by more than a single mechanism. It should be noted that by using the same procedure [25] low levels of the attempt-to-escape frequency (ν_0_ = 2 × 10^5^ s^−1^) and the trap depth (*E*_t_ ≈ 0.36 eV) have also been found for polypropylene [28].

**Figure 16:**
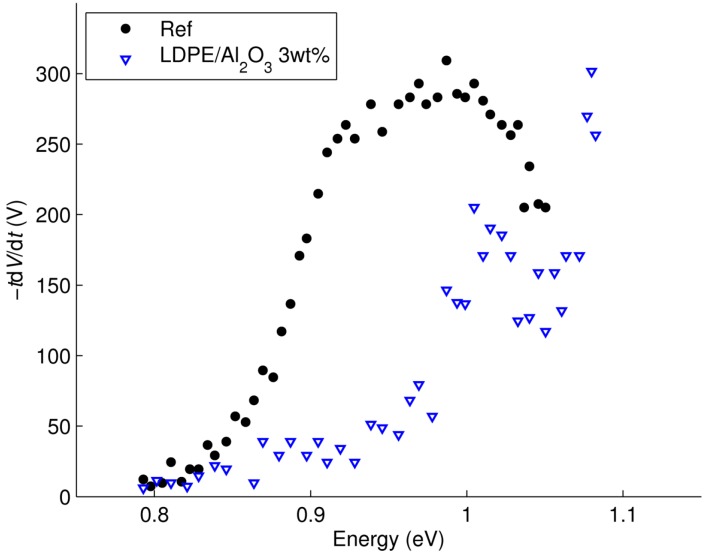
Trap energy distributions in reference LDPE and its nanocomposite.

In Figure 16, the energy distributions of traps are compared for the reference LDPE and LDPE/Al_2_O_3_ nanocomposite by assuming the attempt-to-escape frequency ν_0_ ≈ 6 × 10^12^ s^−1^. For the reference material, the distribution is characterized by a peak at ~1 eV, which may be associated with trapping centra revealed by measurements of thermally stimulated currents (TSC) and attributed to physical defects in amorphous-crystalline interfaces and in crystalline region of PE [40]. For the nanofilled material, the image of trap distribution shifts to deeper trap energy. A shoulder is also found at ~1 eV, suggesting an identical origin as in reference LDPE. In addition, the trap energy distribution of the nanocomposite implies a peak arising outside of the measurement window (at time exceeding 4.2 × 10^5^ s) that can be associated with a deeper trap level (*E*_t_ > 1.1 eV). The appearance of this trapping level is most likely caused by the presence of nanofillers in the material; in particular in the interfacial region between nanofiller particles and the polymer. The energy depth of the trap may be as high as 2 eV, as revealed by TSC measurements on LDPE/MgO nanocomposite [41].

### 3.6. Current Density Deduced from SPD Measurements

During SPD measurement under open circuit configuration, the total current density in elementary volume of dielectric is zero

(7) $$j(x,t)+\frac{\partial \left(\varepsilon E\left(x,t\right)\right)}{\partial t}=0$$

where *j*(*x*,*t*) is space- and time-dependent conduction current density and the second term represents displacement current, ε being the real part of material permittivity, *E* stands for electric field. The externally measurable conduction current density through the insulation can be defined as

(8) $$J(t)=\frac{1}{L}\int_{0}^{L}j(x,t)dx$$

where *L* is sample thickness. By substituting Equation (7) into Equation (8) and noting the flat response of ε on frequency for LDPE and its nanocomposites [10], one obtains

(9) $$J(t)=-\frac{\varepsilon }{L}\int_{0}^{L}\frac{\partial E\left(x,t\right)}{\partial t}dx=-\frac{\varepsilon }{L}\frac{dV(t)}{dt}$$

Equation (9) establishes a relationship between the conduction current density in SPD experiments and the decay rate of the measured potential. The current density is thus calculated and its dependence on electric field is examined in this section. Here the average magnitude of the electric field induced in the insulation *E* = *V*/*L* is used.

**Figure 17:**
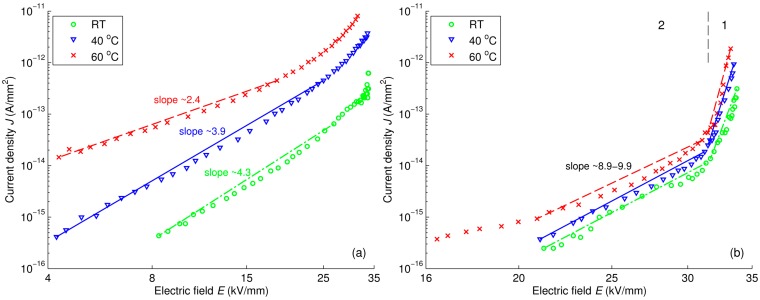
Log-log plot of *J* *vs.* *E* for reference LDPE (**a**); and LDPE/Al_2_O_3_ 3wt % nanocomposite (**b**) at various temperatures. Regions 1 and 2 in figure (**b**) are featured by different slopes of the dependencies.

A log-log plot of the current density *versus* electric field in reference LDPE presented in Figure 17a indicates that factor *m* in the dependency *J* ∝ *E^m^* decreases with temperature. Since *m* > 2, the conduction current in reference LDPE is most likely governed by the space charge limited current (SCLC) mechanism for materials with traps, see Equation (10) [42], rather than the SCLC in trap-free materials described by the Mott-Gurney square law, see Equation (11) [43]:

(10) $$J\propto \frac{V^{l+1}}{L^{2l+1}}$$

(11) $$J=\frac{9}{8}\varepsilon \mu \frac{V^{2}}{L^{3}}$$

**Figure 18:**
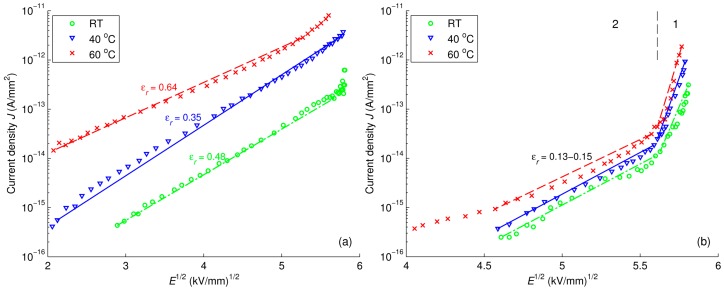
Schottky plot for reference LDPE (**a**); and LDPE/Al_2_O_3_ 3 wt % nanocomposite (**b**) at various temperatures. Regions 1 and 2 in figure (**b**) are featured by different slopes of the dependencies.

**Figure 19:**
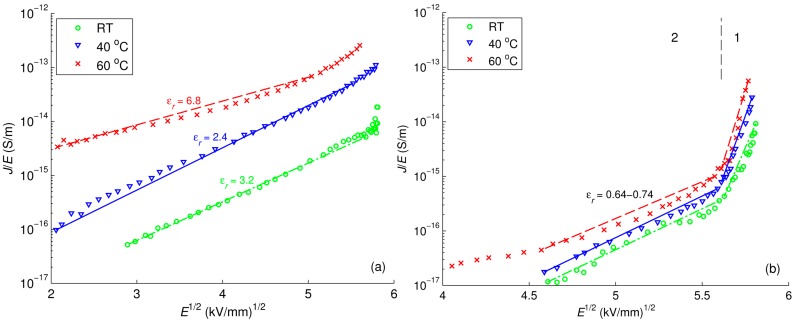
Poole-Frenkel plot for reference LDPE (**a**); and LDPE/Al_2_O_3_ 3 wt % nanocomposite (**b**) at various temperatures. Regions 1 and 2 in figure (**b**) are featured by different slopes of the dependencies.

In Equation (10), factor *l* = *T_C_*/*T*, where *T_C_* is the characteristic temperature of the proposed exponential distribution of trap density [42]. Further, Schottky and Poole-Frenkel plots for reference LDPE are illustrated in Figure 18a and Figure 19a, respectively. As seen, the magnitudes of the relative permittivity used to get best fit (provided in the curves) are quite different from the value 2.3 commonly reported for PE. This fact indicates that neither Schottky injection mechanism nor Poole-Frenkel mechanism satisfactorily explain the behavior of the conduction current density *J* at all considered temperatures. The change of the mechanism governing the conduction in LDPE with temperature has been noted in [44], where Schottky injection has been found to dominate at room temperature, but not at elevated ones.

The field dependencies of current density in LDPE/Al_2_O_3_ nanocomposite are illustrated in Figure 17b, Figure 18b and Figure 19b, whereas the calculated results for LDPE/MgO nanocomposite are provided in supplementary materials (Figures S4–S6). The derived parameters *m* and ε_r_ of these dependencies are provided in Table 2 for comparison. For both nanocomposites, the current density curves show a knee point at which the slopes change, and hence, the characteristics can be divided into two regions as indicated in the figures. It is noteworthy that the time corresponding to the observed knee point is close to the transit time calculated by using the hole mobility deduced in Section 3.4. Thus, the rapid decrease of current densities within the first region can be explained by a transient process followed the charge injection at the air-insulation surface. As the injected charges reach the counter electrode, the field dependence of current densities become less pronounced, as shown in the second region. Parameters *m* and *ε*_r_ are thus calculated only in the latter region for avoiding the effect of the transient process at the initial stage. As seen, the power factor *m* in the dependency *J* ∝ *E^m^* is significantly higher for both nanocomposites as compared to that of reference LDPE. SCLC mechanism followed Equation (10) appears to be the dominating conduction mechanism in the nanocomposites. In contrast, both Schottky injection mechanism and Poole-Frenkel mechanism cannot fully explain the experimental data of the nanofilled materials under consideration. This topic therefore requires further investigation.

## 4. Conclusions

Charge transport in low-density polyethylene (LDPE) filled with nanoparticles of alumina (Al_2_O_3_) and magnesia (MgO) as well as the unfilled counterpart was investigated by means of conductivity and surface potential decay (SPD) measurements. As compared to the pure polymer case, a remarkable reduction in dc conductivity was found for both LDPE nanocomposites at filler content of 3 wt %. Results of SPD measurements on multilayered samples strongly suggest that (a) charge injection at the air-insulation interface and the transport of injected charges are dominating in decay process; and (b) positive charges are prevailing in LDPE. Based on these, mobility of holes in the considered materials has been deduced by using measured data on single-layered samples. The reduced mobility of charge carriers and the increased trap depth obtained in nanocomposites are closely correlated with the weakened charge transport, and hence, decreased dc conductivity of the nanofilled materials. Additionally, by using the measured current density and the calculated charge mobility, lower activation energies were obtained for nanocomposites compared to unfilled LDPE, indicating weaker temperature dependencies of the studied properties in nanofilled dielectrics. The field dependency of the current density derived from SPD measurements was analyzed, showing that the conduction mechanisms in studied materials are strongly affected by presence of nanofillers and temperature. The obtained experimental results are further utilized for computer simulations of charge transport in LDPE and its nanocomposites which are presented in the second part of the work.

## Figures and Tables

**Table 1:** Activation energies (in eV) of dc current density and carrier mobility for reference LDPE and its nanocomposites.

Materials	Derived from Current Density	Derived from Charge Mobility
LDPE	0.85	0.56
LDPE/Al_2_O_3_ 3 wt %	0.43	0.42
LDPE/MgO 3 wt %	0.35	0.17

**Table 2:** Calculated parameters in characteristics of *J* *vs.* *E*. Note that the obtained values of *ε*_r_ below 1 do not have physical significance.

Characteristics	Calculated Parameters	LDPE			LDPE/Al_2_O_3_ NC			LDPE/MgO NC		
		RT	40 °C	60 °C	RT	40 °C	60 °C	RT	40 °C	60 °C
*J* ∝ *E^m^*	*m*	4.3	3.9	2.4	9.8	9.9	8.9	11.5	9.6	5.6
Schottky	ε_r_	0.48	0.35	0.64	0.15	0.13	0.14	0.12	0.14	0.30
Poole-Frenkel	ε_r_	3.2	2.4	6.8	0.74	0.64	0.72	0.56	0.67	1.76

## Supplementary Materials

The supplementary materials can be found at www.mdpi.com/2073-4360/8/3/87/s1.
