# Supplementary material for: Charge Transport in LDPE Nanocomposites Part I—Experimental Approach
Source: Polymers (Basel). 2016 Mar 16;8(3):87. doi: 10.3390/polym8030087 (PMC6432595; doi:10.3390/polym8030087)
Supplement: Supplementary file 1 [file polymers-08-00087-s001.pdf]

Supplementary Materials: Charge Transport in LDPE Nanocomposites Part I—Experimental Approach

Anh T. Hoang, Love Pallon, Dongming Liu, Yuriy V. Serdyuk, Stanislaw M. Gubanski and Ulf W. Gedde

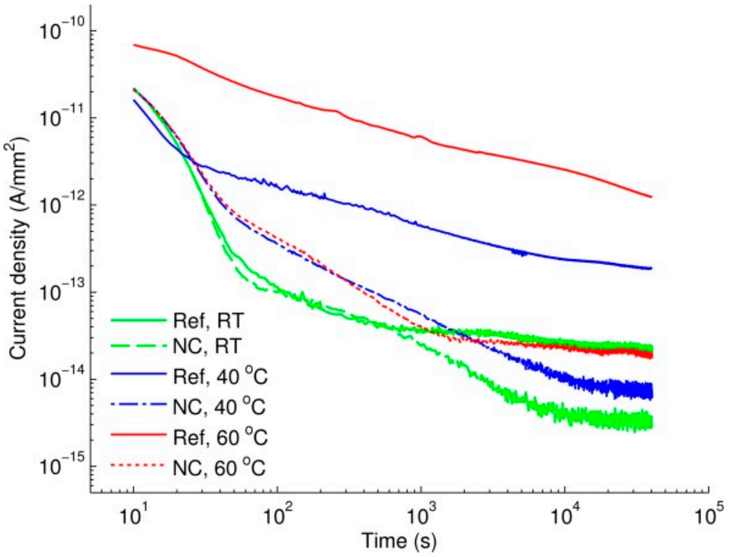

Figure S1. Densities of charging currents as functions of time measured at room temperature (RT) ~20–22 °C, 40 °C, and 60 °C for the reference LDPE (Ref) and LDPE/MgO 3 wt % nanocomposite (NC).

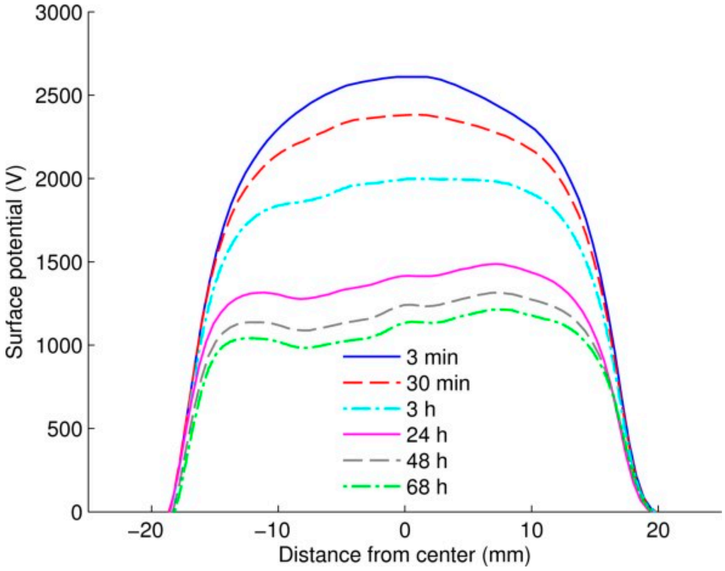

Figure S2. Distribution of surface potential during potential decay measurement on LDPE/MgO 3wt % nanocomposite at 60 °C.

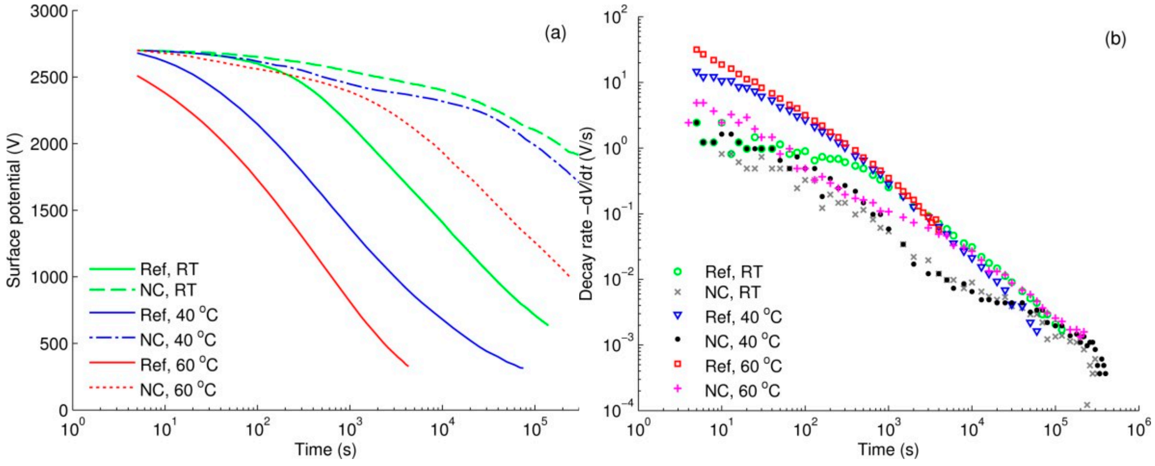

Figure S3. Measured surface potentials (**a**); and calculated decay rates (**b**) for reference LDPE (Ref) and LDPE/ MgO 3 wt % nanocomposite (NC) at different temperatures.

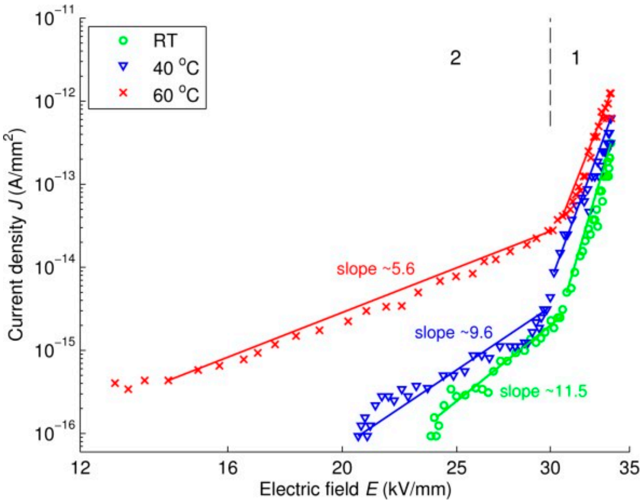

Figure S4. Log-log plot of *J* vs *E* for LDPE/MgO 3 wt % nanocomposite at various temperatures.

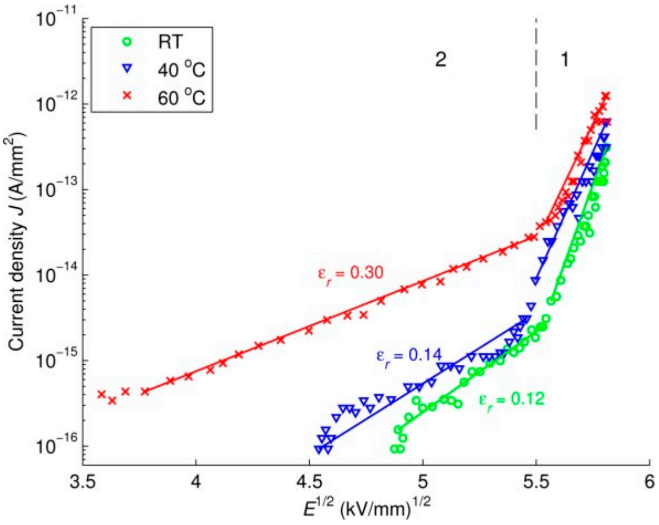

Figure S5. Schottky plot for LDPE/MgO 3 wt % nanocomposite at various temperatures.

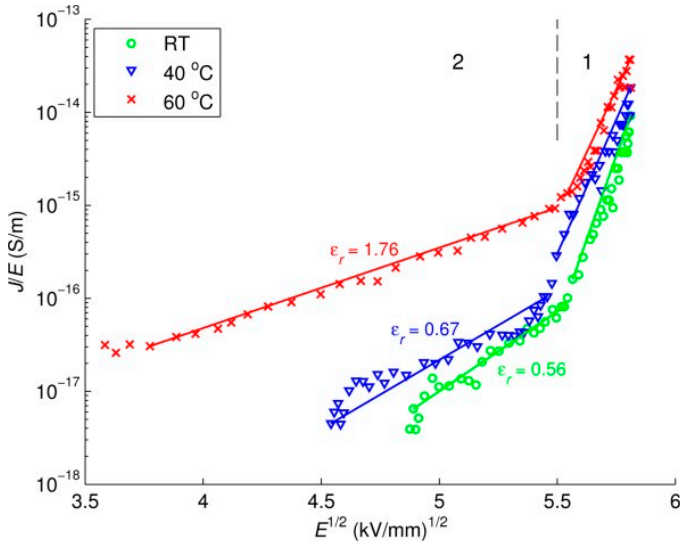

Figure S6. Poole-Frenkel plot for LDPE/MgO 3 wt % nanocomposite at various temperatures.

© 2016 by the authors; licensee MDPI, Basel, Switzerland. This article is an open access article distributed under the terms and conditions of the Creative Commons by Attribution (CC-BY) license (http://creativecommons.org/licenses/by/4.0/).
